# 非小细胞肺癌术后复发风险预测研究进展

**DOI:** 10.3779/j.issn.1009-3419.2026.101.21

**Published:** 2026-08-20

**Authors:** Shihang ZHANG, Haitao YANG, Zhibin HUANG, Baosong XIE

**Affiliations:** 350001 福州，福建医科大学省立临床医学院，福州大学附属省立医院呼吸与危重症医学科; Provincial School of Clinical Medicine, Fujian Medical University; Department of Pulmonary and Critical Care Medicine,Provincial Hospital of Fuzhou University, Fuzhou 350001, China

**Keywords:** 肺肿瘤, 人工智能, 液体活检, 预测模型, Lung neoplasms, Artificial intelligence, Liquid biopsy, Predictive model

## Abstract

肺癌是全球范围内发病率和死亡率最高的恶性肿瘤，其中非小细胞肺癌（non-small cell lung cancer, NSCLC）约占85%。根治性手术切除是可切除NSCLC患者获得长期生存的主要手段，但仍有部分患者出现术后复发，影响预后转归。因此，精准预测术后复发风险、筛选高危人群并制定个体化辅助治疗及随访策略，对改善患者预后具有重要意义。近年来，随着人工智能与液体活检等技术的快速发展，NSCLC术后复发风险预测正逐步迈向精细化与个体化。本文综述传统模型、人工智能模型及液体活检技术在NSCLC术后复发预测中的应用现状，分析其优势与局限，并对未来研究趋势进行展望。

## Abstract (en)

Lung cancer remains the malignant tumor with the highest incidence and mortality rates worldwide, with non-small cell lung cancer (NSCLC) accounting for approximately 85% of cases. Radical surgical resection serves as the primary curative modality for patients with resectable NSCLC; however, postoperative recurrence persists in a subset of patients, thereby compromising long-term survival outcomes. Consequently, the precise prediction of postoperative recurrence risk, identification of high-risk populations, and formulation of individualized adjuvant therapy and surveillance strategies are of paramount importance for improving patient prognosis. In recent years, fueled by the rapid evolution of artificial intelligence (AI) and liquid biopsy technologies, the prediction of postoperative recurrence in NSCLC has progressively transitioned toward greater precision and personalization. This review summarizes the current applications of traditional predictive models, AI-based models, and liquid biopsy techniques in forecasting NSCLC recurrence following surgery, evaluating their respective strengths and limitations. Furthermore, it provides insights into future research trajectories.

肺癌是全球范围内发病率和死亡率均位居首位的恶性肿瘤^[1]^，非小细胞肺癌（non-small cell lung cancer, NSCLC）约占85%，其中肺腺癌（lung adenocarcinoma, LUAD）和肺鳞状细胞癌（lung squamous cell carcinoma, LUSC）是最常见的亚型^[2]^；对于可切除NSCLC患者，根治性手术切除是获得长期生存的基础，但术后仍有部分患者复发^[3]^。这一问题不仅影响患者生存质量和生存期，也给家庭和社会带来了沉重负担。因此，如何在根治性切除术后识别高复发风险患者，实施积极辅助治疗与严密随访策略，对改善患者预后有重要价值。目前，有研究^[4,5]^报道了NSCLC术后复发相关的各种高危因素，涵盖了人口学特征（如年龄、性别）、影像学表现（如肿瘤大小、肿瘤密度）、血液标志物（如癌胚抗原）、病理学特征（如分化程度、脉管癌栓、脏层胸膜侵犯、气腔播散）、手术相关因素（如术式、淋巴结清扫程度）及基因分子特征（如驱动基因突变状态、肿瘤突变负荷）等多个方面。对这些高危因素的深入认识与梳理，为后续复发风险预测模型的构建奠定了坚实的理论基础。

近年来，随着人工智能（artificial intelligence, AI）、生物技术的飞速发展，为NSCLC术后复发风险预测开辟了新路径。这些新兴技术凭借其强大的自动特征学习能力、高维数据的整合分析优势或超高灵敏度的分子探测能力，有望从更深层次揭示肿瘤复发的内在机制，并构建出性能更优的预测模型^[6,7]^。本文旨在系统回顾传统模型、AI模型、液体活检技术在预测NSCLC术后复发风险的研究现状，并展望未来发展趋势。

## 1 传统模型

传统临床预测模型主要包含*Cox*比例风险回归与*Logistic*回归模型。该类模型通过整合临床变量，建立自变量与复发结局之间的定量关联，并以风险比（hazard ratio, HR）或优势比（odds ratio, OR）的形式输出各因素的效应值^[8]^。在此基础上，研究者依据预设的阈值对变量进行筛选，构建最终预测模型，并进一步通过列线图实现可视化，直观展示各变量的评分权重及个体化的预后风险。其具有临床可解释性强、计算简便等特点^[9]^。纵观现有传统临床预测模型的研究，可以从两个角度切入：变量维度拓展和适用情景细分。变量维度拓展体现在变量来源的广谱性与组合策略的多样性。预测因子涵盖人口学特征、影像学表现、病理学特征、外周血指标、基因突变信息等。而组合策略的多样性则反映在研究者根据临床场景与数据特点，灵活采用多种整合模式。

除上述提到的传统临床变量外，近年来血液复合指标和影像组学进一步拓展了变量来源。血液复合指标是指基于常规外周血检测数据构建的综合参数，例如中性粒细胞与淋巴细胞比值（neutrophil-to-lymphocyte ratio, NLR）、血小板与淋巴细胞比值（platelet-to-lymphocyte ratio, PLR）等^[10]^。影像组学是指通过从影像图片中提取定量特征，包括形状、纹理、灰度分布等，将视觉图像转化为高通量的计算数据，以刻画感兴趣区域的影像表型信息^[11]^。这两者作为二次衍生变量，分别能够反映人体的全身炎症、肿瘤局部的表型异质性与微环境特征^[10,12]^。

已有多项研究在变量筛选阶段便引入血液复合指标及影像组学特征，且该类变量在建模过程中常被证实具有统计学意义，保留于最终预测模型中，甚至与传统临床变量整合后，有助于提升模型预测性能，如附表1^[13-22]^（http://www.lungca.org/files/2026-06-0003/supp-2026-06-0003.pdf）部分展示。其中Hao等^[16]^通过对918例IB期NSCLC患者分析，系统构建了包含术前、术后及差值变化的营养与免疫复合指标预测模型。虽然该模型能充分利用围术期血液学指标来预测NSCLC术后复发，但是各模型C指数仅为0.676、0.604、0.613，区分效能一般。Li等^[14]^在含微乳头成分的IA期LUAD研究中，将影像组学与微乳头占比、气腔播散及癌胚抗原等临床高危因素整合，模型预测LUAD术后3、5、7年的无病生存期（disease-free survival, DFS）展现优秀的区分能力，受试者工作特征曲线下面积（area under the curve, AUC）分别为0.817、0.805、0.811。此外，Chen等^[15]^在628例IA期患者中系统比较了国际肺癌研究协会（International Association for the Study of Lung Cancer, IASLC）分级模型、影像组学模型、联合模型在LUAD术后预测死亡和复发风险方面的价值，发现联合模型表现最优（C指数=0.701）。

适用情景细分体现在依据关键临床病理或治疗特征等将人群限制在相对同质的亚组内，以排除混杂偏倚。例如，可区分不同肿瘤原发灶-淋巴结-转移（tumor-node-metastasis, TNM）分期、组织学类型、术式等，如附表1^[13-22]^（http://www.lungca.org/files/2026-06-0003/supp-2026-06-0003.pdf）“适用情况（Applicability）”部分展示。在分期维度上，模型多聚焦于I期等较早期阶段^[13-16,20]^，主要缘于TNM分期本身是复发的强预测因子，若将其纳入模型往往会主导预测结果，从而稀释其他潜在重要协变量的贡献度。在病理维度上，进一步细化至LUAD，并延伸至含微乳头成分或伴表皮生长因子受体（epidermal growth factor receptor, *EGFR*）突变等特定分子亚型^[13-15,18,20]^；在治疗维度上，则涵盖了全肺切除或单孔腔镜等不同术式的评估^[19,22]^。其中，Zhang等^[17]^基于逾5000例患者的多中心大样本队列，构建了针对完全切除NSCLC患者不同解剖部位复发风险的网页列线图模型。该研究不仅针对复发进行总体预测，更系统地对胸腔、颅脑、骨骼等特定部位分别建模，还引入了条件风险概念，能够结合患者末次阴性随访时间动态更新复发概率，更贴合临床实际监测场景，但研究者未进行系统的临床获益分析，且“个体化术后随访计划”主要体现在部位风险分层方面，缺乏具体的随访方案指导。

尽管传统临床预测模型在NSCLC术后复发评估中发挥了重要作用，但从技术角度看，其局限性仍体现在以下几个方面：（1）传统模型多基于*Logistic*或*Cox*回归的线性假设，其难以刻画肿瘤复发过程中多因素间复杂的非线性交互机制；（2）传统模型多基于静态数据评估，缺乏对围术期及术后复查结果及治疗的时序建模能力；（3）在面对多中心、多组学整合的数据集时，传统统计方法在计算效率、高维数据处理等方面存在瓶颈^[23,24]^。

## 2 AI模型

随着科学技术的快速发展，AI正深刻改变着医学研究与临床实践的模式。AI模型凭借其非线性拟合能力与高维特征学习优势，逐渐成为突破传统统计方法局限的重要工具。目前，应用于临床预测模型的方法主要涵盖传统机器学习与深度学习两大类^[25]^。

### 2.1 传统机器学习模型

传统机器学习通常指以人工特征和浅层为主要特征的一类数据驱动算法。它通过优化特定性能准则从数据中学习输入到输出的映射，并用于未知样本的预测与决策。其“传统”主要相对于深度学习，前者依赖人工特征构建，表示能力受限于特征设计与浅层结构；后者则可通过多层网络自动学习分层特征。与传统预测模型相比，传统机器学习通常不强制预设变量间线性关系或特定分布形式，因而能更灵活地捕捉复杂非线性规律与高阶交互^[26]^。传统机器学习在医学预测建模中的应用通常可按学习方式划分为监督学习、无监督学习与半监督学习三大类。其中，监督学习因可利用已有的临床诊断或标签，成为疾病风险预测、疗效评估与预后分层的主流方法。其代表性算法包括支持向量机（support vector machine, SVM）、随机森林（random forest, RF）、极端梯度提升树（extreme gradient boosting, XGBoost）等^[27]^。

Kinoshita等^[28]^基于影像、病理变量及围术期血液检查，利用XGBoost算法构建NSCLC术后预后模型，其预测5年DFS的AUC达0.890，显著优于单一TNM分期。此外，Lin等^[29]^采用堆叠算法融合多种机器学习模型，构建NSCLC术后2年内早期复发预测工具，外部验证集AUC达到0.804。上述研究是通过大样本、临床常规变量构建的预测模型，均展现了优秀的预测效果。值得注意的是，尽管前文提及部分影像组学研究仍沿用传统统计模型，但鉴于影像组学数据具备的高维度、小样本特性，传统模型易面临过拟合，而机器学习算法在处理此类复杂特征空间上更具鲁棒性。因此，近年多数学者更倾向于融合影像组学特征与临床变量构建多模态模型^[30-34]^。Ho等^[30]^通过SVM算法整合术前计算机断层扫描（computed tomography, CT）影像组学与临床变量，构建术前和术后模型，其AUC分别为0.754和0.844；Lin等^[31]^利用融合双期增强CT的瘤内与瘤周放射组学特征及临床病理参数，内部验证集AUC高达0.883；张地等^[32]^则基于自适应提升算法（Adaboost），将平扫CT影像组学特征与年龄、吸烟史等临床风险因素融合，对比单纯的临床模型（AUC=0.806）和影像模型（AUC=0.813），融合模型（AUC=0.852）有更加优秀的判别能力。

其中，Amini等^[33]^最大化利用正电子发射型计算机断层显像（positron emission computed tomography, PET）/CT全扫描范围内多器官信息，提取了术前PET/CT中19个受累及“假定健康”器官的影像组学特征，然后分别构建临床病理、肿瘤区域及多器官影像组学的单源一级模型，再将各模型输出的风险得分作为高阶特征输入模型进行二次学习。结果显示，多源融合后的模型C指数达0.731，优于单一临床病理模型（0.670）和影像组学模型（0.650）。Janik等^[35]^未进行常规变量特征筛选，而是将诊断变量、手术变量、术后治疗变量进行逐步整合并分次建模，构建了RF、GDBT（gradient boosting）等表格机器学习模型及基于知识图谱的图嵌入模型（ComplEx-N3），其中随机森林模型表现最佳（AUC=0.813）。创新性地通过构建知识图谱，将异构临床数据转化为结构化网络，并采用图嵌入模型进行复发预测。但该研究未对复发时间进行限定，将术后不同时间窗的复发事件视为同质结局，忽略了早期与远期复发的异质性。Wang等^[34]^构建了融合术前CT影像组学与N分期的预测模型，比较逻辑回归、SVM、RF及XGBoost等算法。结果显示，经过特征筛选后传统逻辑回归模型却表现最佳，其内部验证集AUC高达0.937；相比之下，部分复杂的非线性算法虽然在训练集上表现优异，但在验证集中出现了明显的性能衰减，提示在样本量有限且特征经严格筛选的场景下，高复杂度模型极易因过拟合而导致泛化性能衰减，此时凭借低方差特性与强可解释性的传统回归模型，反而展现出不可替代的临床价值。各种传统机器学习预测模型信息详见附表2^[28-35]^（http://www.lungca.org/files/2026-06-0003/supp-2026-06-0003.pdf）。

### 2.2 深度学习模型

尽管传统机器学习算法在结构化数据预测中展现出良好性能，但其效能取决于研究者能否提炼出最具判别力的特征，而且，还面临图像标注成本高昂、过程耗时等问题^[36]^。深度学习是一种基于深度神经网络的AI算法，能通过层级化的非线性变换，自动从大规模数据中逐层提取特征。在临床预测场景下，深度学习尤其适用于非结构化或高维数据，如影像图片、病理切片、电子病历等，能够自动挖掘数据内部复杂的空间、时序或语义关联。其涵盖多种算法架构，各自有不同的适用场景：如适合处理图像的卷积神经网络（convolutional neural network, CNN）、适合处理纵向随访的循环神经网络（recurrent neural network, RNN）、适合捕捉长序列电子病历的自注意力模型等^[25,37]^。

在深度学习驱动的影像组学领域，研究者们针对胸部CT二维与三维特征提取进行了探索。Lian等^[38]^尝试将视觉Transformer引入早期NSCLC复发预测，利用自注意力机制提取肿瘤全局特征，在外部验证集中AUC值为0.70。更进一步，Fu等^[39]^开发的3D自适应密集连接网络（3D-AdaDense）则弥补了前述二维网络难以充分刻画肿瘤立体的局限，在内部验证中将C指数提升至0.850，显著优于传统临床模型（C指数=0.760）。相较之下，病理组学则聚焦于全切片图像（whole slide image, WSI）的微观特征挖掘，从中量化肿瘤生长模式与免疫浸润状态。其中，Chen等^[40]^基于图注意力多实例神经网络构建的模型在多中心验证中将LUAD患者划分为预后显著差异的高低风险组（HR=1.95, *P*<0.001），其WSI风险评分与TNM分期、IASLC分级联合后C指数提升至0.811。另外，Li等^[41]^基于CAMEL2弱监督混合神经网络，整合病理WSI与临床数据，预测5年DFS的C指数为0.775。该模型可将患者划分为预后显著不同的高、低风险组，高风险组相较低风险组的复发或死亡风险显著升高（HR=4.85, *P*<0.0001）。

此外，有部分学者对影像组学和病理组学融合进行了探索。Vaidya等^[42]^构建了结合CT放射组学与病理组学特征的多模态模型，并借助U-Net深度学习辅助病理图像分割，其中对NSCLC患者术后复发预测的复合模型C指数达0.710。Lin等^[43]^利用nnU-Net与Hover-Net深度学习架构分别对胸部CT和病理切片进行分割，构建的复合模型性能在多个外部中心表现优秀（中心1：C指数=0.719，中心2：C指数=0.711）。但是这两项研究是利用深度学习作为工具来辅助图像分割，并非真正的“端到端”特征自动提取。

除上述基于影像和病理的深度学习广泛应用外，有学者引入自然语言处理方法，对长文本数据进行了自动提取及纵向时序分析。Jung等^[44]^开发的RADAR CARE模型利用Transformer架构同时编码基线临床病理数据、纵向实验室指标以及影像文本报告，实现了针对患者术后1年内复发风险的实时动态预警（AUC=0.854）。

深度学习在NSCLC术后预测复发方面仍需要拓展：多数报道仅体现为放射组学或病理组学特征与临床变量的结合，或深度学习仅仅用于图像分割，真正实现影像与病理双模态特征全自动提取的模型仍较为匮乏，在一定程度上限制了模型对肿瘤宏观形态与微观表型异构性的同步捕捉能力。另外，在医学图像相关的深度学习中，未联合临床变量的模型区分度性能多数一般，如附表3^[38-44]^（http://www.lungca.org/files/2026-06-0003/supp-2026-06-0003.pdf）“区分度（Discrimination）”部分展示。因为医学图像数据通常只有千百例，而深度学习模型有数百万参数，参数远多于数据，模型有足够容量记住每张图，导致泛化能力下降。

## 3 液体活检

NSCLC术后患者的长期随访管理主要依赖于影像学与血清肿瘤标志物的动态复查。然而，这种复查策略存在一定时间滞后性，影像学手段往往仅在肿瘤负荷达到一定阈值或引发结构性改变时方能检出，此时患者可能已丧失最佳干预窗口^[7]^。液体活检技术是一种非侵入性的新型检测技术，借助对血液、唾液、尿液等体液样本的分析来检测目标信号。在肿瘤诊疗中，其检测对象涵盖循环肿瘤DNA（circulating tumor DNA, ctDNA）、循环肿瘤细胞（circulating tumor cells, CTCs）、外泌体、循环肿瘤RNA（circulating tumor RNA, ctRNA）等，能够对NSCLC患者术后识别微小残留病灶（minimal residual disease, MRD）提供重要价值^[45]^。Shen等^[46]^通过一项纳入34项诊断研究与21项预后研究的荟萃分析，评估了液体活检在早期NSCLC中的临床效用。研究发现术后MRD阳性是复发的独立危险因素（HR=4.95），且分子复发平均较影像学进展提前约179天。

近年来，液体活检领域呈现明显的分化，ctDNA逐渐成为研究热门。相比之下，CTCs虽能提供完整的活细胞及转移生物学证据，但其检出率受限于早期肿瘤释放量极低，且样本处理时效苛刻、富集技术平台间差异大，至今未能形成可规模化的临床转化路径。外泌体、ctRNA则因分离纯化缺乏统一标准、标志物特异性不足、检测流程复杂等问题，尚处于方法学探索阶段^[47,48]^。目前有多项国内外前瞻性队列研究在进行，如附表4^[49-61]^（http://www.lungca.org/files/2026-06-0003/supp-2026-06-0003.pdf）展示。

DYNAMIC研究^[49]^是国内首个解析肺癌根治术围术期ctDNA动力学的前瞻性队列，精确测得R0切除后ctDNA中位半衰期仅35 min，并据此确立术后第3天为MRD基线检测最佳时间窗。CALIBRATE研究^[50]^则是用纵向ctDNA轨迹与复发时间耦合，实现对NSCLC术后复发风险的动态更新预测，联合模型在术后12个月复发预测的AUC达0.890。LumpCure研究^[51]^反转传统“阳性预测复发”思路，以ctDNA纵向阴性定义“潜在治愈”，证实纵向持续MRD不可测者仅3.2%（6/187）复发，阴性预测值（negative predictive value, NPV）高达96.8%，在长程随访中，纵向MRD不可测人群的NPV仍有93.2%^[52]^。LUNGCA研究^[53]^则基于术前、术后3天及1个月围术期ctDNA监测，发现术后1个月内ctDNA阳性患者复发率高达80.8%，MRD状态是无复发生存期（recurrence-free survival, RFS）缩短的强预测因子（HR=11.1），且在多因素模型中预测的相对贡献超过TNM分期等所有临床病理变量之和。而在长程随访中把ctDNA扩展为覆盖“术前-围术期-辅助治疗-复发后”全节点的动态管理，团队提出并验证“综合治疗后MRD状态”是更优复发预测指标（NPV=90.3%, HR=28.2, *P*<0.001）^[54]^。但以上队列研究采用的都是固定化panel策略，灵敏度较低，且不同panel覆盖的基因突变点位存在较大差异。MEDAL队列^[55]^是全球首个头对头对比ctDNA肿瘤先验个体化策略、肿瘤先验固定化策略、肿瘤未知固定化策略的前瞻性队列。研究发现个体化策略术前灵敏度达41.7%，显著优于肿瘤先验固定策略的21.9%以及肿瘤未知固定策略的17.2%。近期另一项国内前瞻性队列^[56]^同样对比了3种ctDNA-MRD检测策略优劣，在术后1个月监测点，个体化策略检测效能同样显著优于两种固定化策略（灵敏度分别为40.9%、27.3%、13.7%）。

TRACERX研究^[58]^旨在通过多区域肿瘤组织全外显子组测序联合纵向液体活检，系统描绘了肺癌的瘤内异质性演化与临床结局关联，被视为NSCLC进化生物学与MRD研究的里程碑式队列。其中Black等^[58]^定制了中位含1800个靶点的超灵敏个性化ctDNA-panel，术前ctDNA阳性率高达82.7%，并通过2994份纵向血浆样本实现了超高密度的时序监测，首次量化了术后ctDNA的增长斜率，将分子动力学特征与复发解剖模式进行关联，即“慢增长”表型倾向于胸内或脑部孤立复发，而“快增长”表型则预示胸外广泛转移。但是该队列入组时间较早，辅助治疗分析仅纳入含铂方案化疗患者，缺乏靶向及免疫治疗信息。Abbosh等^[59]^基于同一队列，把ctDNA-MRD从临床分层推进到生物学本质探索，发现NSCLC复发并非源于整体肿瘤负荷，而是由术前已获得全基因组加倍和染色体不稳定性的优势亚克隆所驱动。另外，Chemi等^[60]^在通过CellSearch平台对NSCLC引流的肺静脉血进行CTCs捕获，证实CTCs与转移灶同属一支早期播散亚克隆。该类细胞因进化潜能强、更易入血，术后持续检出ctDNA与CTCs即代表该亚克隆的微量残留。综上，TRACERx研究在分子演化、细胞播散、临床分层三方面对NSCLC术后复发进行了开创性实证。

然而，上述队列研究所展示的优异性能仍需审慎解读，因其始终面临技术噪音与生物学异质性的问题。假阴性主要源于如早期肺癌低脱落、检测平台测序深度或亚克隆突变覆盖不足等。假阳性则多由克隆性造血突变混淆、超敏感测序引入的技术噪音等^[47,48]^。此外，现有多数研究受限于单中心、小样本量，缺乏随机对照试验证据来验证“基于MRD状态的干预策略”能否真正转化为生存获益，而非仅仅停留在预后关联层面。

## 4 小结与展望

NSCLC患者在接受根治性切除术后仍面临复发转移风险，故如何高效预测复发风险对指导辅助治疗及随访策略具有重要意义。不同预测模型各有优劣，需综合评估模型特点，并结合实际样本、临床场景等选择。3类主要临床预测模型优缺点详见表1。需要注意的是，基于“TRIPOD+AI”^[27]^与“PROBAST+AI”^[62]^框架，当前NSCLC术后复发预测模型研究在报告完整性、方法学质量方面存在诸多共性缺陷。首先，在样本评估方面，多数研究未进行正式的样本量计算，事件数与变量数之比低于传统回归模型所建议的10:1阈值，而机器学习与深度学习模型对样本量的需求实则更高。其次，NSCLC术后复发率随肿瘤分期变化而呈现显著差异，数据集中阳性事件占比偏低，类别分布不均。模型倾向于以高准确率预测占多数的阴性事件，阳性预测价值受限。在变量处理方面，如数据缺失插补、变量选择依据、变量间多重共线性问题未详细提及。在模型构建及验证方面，多数模型超参数调优报告缺失，研究缺乏独立外部验证等。

**表1:** 3种常用临床预测模型对比

Models	Algorithms	Strengths	Limitations
Traditional models	Logistic, Cox	High interpretability (Nomograms); Well-established statistical inference; Robustness in small samples	Limited capacity for complex nonlinear relationships; Difficulty handling high-dimensional and interaction features; Labor-intensive manual feature engineering
Traditional machine learning models	SVM, KNN, RF, XGBoost, GBDT, NN	Proficient in modeling high-dimensional data and feature interactions; Excellent capacity for complex nonlinear relationships; Well-suited for structured tabular data; High computational efficiency	Moderate interpretability (SHAP); Moderate data demand
Deep learning models	MLP, CNN, RNN, Transformer	Automated feature extraction (imaging, text); Competence in multimodal data integration	Poor interpretability; High data demand; High training and deployment costs

SVM: support vector machine; KNN: k-nearest neighbors; RF: random forest; XGBoost: extreme gradient boosting; GBDT: gradient boosting decision tree; NN: neural network; SHAP: shapley additive explanations; MLP: multi-layer perceptron; CNN: convolutional neural network; RNN: recurrent neural network.

近年来，液体活检技术的快速发展，有效弥补了临床预测模型对早期复发及微小转移灶预测能力的不足。未来研究应着力于三方面突破：一是深化多模态数据融合，纳入临床表格数据、影像图片、病理切片、液体活检等变量，构建系统性的预后预测体系；二是推动算法创新，利用深度学习、注意力机制等技术提升模型对时序性数据与隐匿模式的捕捉能力；三是强化临床转化闭环，通过前瞻性、多中心、大样本、真实世界的研究来验证模型效能，并增强其可解释性，最终形成覆盖术前预警、术后动态监测及干预决策支持的个体化精准管理路径，为实现NSCLC术后复发风险的精准防控提供有力工具。
